# Characteristics and Evolutionary Analysis of Photosynthetic Gene Clusters on Extrachromosomal Replicons: from Streamlined Plasmids to Chromids

**DOI:** 10.1128/mSystems.00358-19

**Published:** 2019-09-10

**Authors:** Yanting Liu, Qiang Zheng, Wenxin Lin, Nianzhi Jiao

**Affiliations:** a State Key Laboratory for Marine Environmental Science, Institute of Marine Microbes and Ecospheres, Xiamen University, Xiamen, People’s Republic of China; b College of Ocean and Earth Sciences, Xiamen University, Xiamen, People’s Republic of China; Dartmouth College

**Keywords:** aerobic anoxygenic photoheterotrophic bacteria, AAPB, photosynthetic gene cluster, PGC, extrachromosomal replicons, ECRs, *Roseobacter* clade

## Abstract

Aerobic anoxygenic photoheterotrophic bacteria (AAPB) represent a bacteriochlorophyll *a*-containing functional group. Substantial evidence indicates that highly conserved photosynthetic gene clusters (PGCs) of AAPB can be transferred between species, genera, and even phyla. Furthermore, analysis of recently discovered PGCs carried by extrachromosomal replicons (exPGCs) suggests that extrachromosomal replicons (ECRs) play an important role in the transfer of PGCs. In this study, 13 *Roseobacter* clade genomes from seven genera that harbored exPGCs were used to analyze the characteristics and evolution of PGCs. The identification of plasmid-like and chromid-like ECRs among PGC-containing ECRs revealed two different functions: the spread of PGCs among strains and the maintenance of PGCs within genomes. Phylogenetic analyses indicated two independent origins of exPGCs, corresponding to PufC-containing and PufX-containing *puf* operons. Furthermore, the two different types of operons were observed within different strains of the same *Tateyamaria* and *Jannaschia* genera. The PufC-containing and PufX-containing operons were also differentially carried by chromosomes and ECRs in the strains, respectively, which provided clear evidence for ECR-mediated PGC transfer. Multiple recombination events of exPGCs were also observed, wherein the majority of exPGCs were inserted by replication modules at the same genomic positions. However, the exPGCs of the *Jannaschia* strains comprised superoperons without evidence of insertion and therefore likely represent an initial evolutionary stage where the PGC was translocated from chromosomes to ECRs without further combinations. Finally, a scenario of PGC gain and loss is proposed that specifically focuses on ECR-mediated exPGC transfer to explain the evolution and patchy distribution of AAPB within the *Roseobacter* clade.

**IMPORTANCE** The evolution of photosynthesis was a significant event during the diversification of biological life. Aerobic anoxygenic photoheterotrophic bacteria (AAPB) share physiological characteristics with chemoheterotrophs and represent an important group associated with bacteriochlorophyll-dependent phototrophy in the environment. Here, characterization and evolutionary analyses were conducted for 13 bacterial strains that contained photosynthetic gene clusters (PGCs) carried by extrachromosomal replicons (ECRs) to shed light on the evolution of chlorophototrophy in bacteria. This report advances our understanding of the importance of ECRs in the transfer of PGCs within marine photoheterotrophic bacteria.

## Abstract (precis)

The evolution of photosynthesis was a significant event during the diversification of biological life. Aerobic anoxygenic photoheterotrophic bacteria (AAPB) share physiological characteristics with chemoheterotrophs and represent an important group associated with bacteriochlorophyll-dependent phototrophy in the environment. Here, characterization and evolutionary analyses were conducted for 13 bacterial strains that contained photosynthetic gene clusters (PGCs) carried by extrachromosomal replicons (ECRs) to shed light on the evolution of chlorophototrophy in bacteria. This report advances our understanding of the importance of ECRs in the transfer of PGCs within marine photoheterotrophic bacteria.

## INTRODUCTION

Aerobic anoxygenic photoheterotrophic bacteria (AAPB) are chlorophototrophic members of the *Proteobacteria* phylum that contain bacteriochlorophyll *a* and are widely distributed in euphotic ocean environments (1–7). AAPB account for up to 10% of the total bacterial communities in the upper ocean layers and play crucial roles in carbon and energy cycling (8–10). AAPB are facultatively phototrophic, using light as an alternative energy source, which thereby reduces the respiration of organic carbon from marine primary production by ∼2.4% to ∼5.4% (4). In addition to their ecological significance, physiological and genomic analysis of AAPB has indicated their important potential to inform on the evolution of photosynthesis (11–13). AAPB are hypothesized to have evolved after widespread oxygenation of Earth’s atmosphere ∼2.4 to ∼2.3 billion years (Ga) ago (14, 15). Purple photosynthetic bacteria are one such group of anaerobic phototrophs that are thought to represent the ancestral lineage to AAPB due to the high similarity of their photosynthetic apparatuses (13, 16, 17). However, unlike the purple photosynthetic bacteria that grow in the absence of oxygen and are mainly autotrophic, AAPB are aerobic heterotrophs that perform phototrophy as an auxiliary energy conservation strategy, which can contribute up to 20% of their total cellular metabolic energy demands (6, 18). Phylogenetic analysis of the 16S rRNA gene indicates that the distribution of AAPB is scattered throughout the *Proteobacteria* and that they are closely related to nonphototrophic bacteria and purple nonsulfur bacteria within the *Proteobacteria* (12, 13, 18).

Similarly to purple photosynthetic bacteria, photosynthetic gene clusters (PGCs) are found in AAPB. The large PGC superoperon is approximately 35 to 50 kb in length and contains the approximately 40 genes required for the biosynthesis of bacteriochlorophyll, carotenoids, photosynthetic reaction complexes, and light harvesting complexes, in addition to other regulatory functions (19–22). PGC gene sequences are highly conserved, and some essential genes within PGCs (e.g., *pufL*, *pufM*, and *bchY*) are typically used as molecular markers in phylogenetic analyses to classify phototrophic *Proteobacteria* and study their ecological diversity and evolutionary relationships (5, 22–25).

Previous comparisons of closely related strains have demonstrated that PGCs can be lost from bacterial genomes. For example, *Citromicrobium* sp. JL1363 lost PGCs from its genome during its evolutionary history and is now reliant solely on heterotrophy (26). Furthermore, PGCs can be transferred between proteobacterial strains and among distantly related phyla. For instance, although *Erythrobacter* sp. AP23 shares 99.5% 16S rRNA gene sequence identity with *Erythrobacter* sp. LAMA915, only the former strain contains a PGC within its genome (23). Intriguingly, the PGC of *Erythrobacter* sp. AP23 is closely related to that of *Citromicrobium* (23). Gemmatimonadetes is a novel phototrophic phylum and has been suggested to acquire its PGC from phototrophic bacteria of the *Proteobacteria* via a horizontal gene transfer (HGT) event (25).

Members of the *Roseobacter* clade are important ecological generalists within marine ecosystems, and many are AAPB (27–29). Inconsistencies within phylogenetic topologies between the 16S rRNA gene and PGCs indicate that HGT events of PGCs have occurred among members of this clade (30–33). Of note, PGC-containing extrachromosomal replicons (ECRs) have been identified within six strains of the *Roseobacter* clade, further indicating the possibility of HGT of PGCs among these species (33). ECRs are mobile genetic elements that carry many essential genes involved in metabolism that are essential for rapid adaptation to changing environments (34–36). Therefore, it is likely that PGCs can also be transferred via ECRs. However, a limited number of studies have focused on the role of ECRs in the HGT of PGCs (31, 33).

In this study, we analyzed 13 *Roseobacter* clade genomes that contain PGCs carried by extrachromosomal replicons (exPGCs). Phylogenetic and structural analyses of the exPGCs, in addition to comparisions against chromosomal PGCs (cPGCs), were used to elucidate the potential role of ECRs in the transfer of PGCs among *Roseobacter* clade species.

## RESULTS AND DISCUSSION

### General features of the 13 *Roseobacter* clade strains.

Thirteen *Roseobacter* clade strains carried PGC-containing ECRs and were affiliated with seven different genera: *Tateyamaria*, *Jannaschia*, *Sulfitobacter*, *Roseobacter*, *Oceanicola*, *Shimia*, and *Nereida* (Table 1). The genome sizes of the 13 strains ranged from 2.89 to 4.75 Mb, while the average level of genomic GC content ranged from 54.0% to 65.5%. Most strains contained more than two ECRs, and the largest ECR among the 13 strains was a 185-kb PGC-containing ECR in *Sulfitobacter* sp. AM1-D1. The 13 identified exPGCs ranged in size from 41.3 to 54.0 kb, and their average GC contents were consistent with their respective chromosomes. All PGC-containing ECRs harbored DnaA-like I replication systems except for that of Sulfitobacter guttiformis, which exhibited a RepB-III type system.

**TABLE 1:** Genomic information for 13 *Roseobacter* strains carrying exPGCs

Genus	Strain	Genome size (Mb)	No. of contigs	Genome GC content (%)	Phototrophic plasmid size (kb)	PS size (kb)^*a*^	Phototrophic plasmid GC content (%)	PGC GC content (%)	Isolation source
*Tateyamaria*	*Tateyamaria* sp. ANG-S1	4.43	33	60.6	139.0	53	61.37	62.59	Accessory nidamental gland
	*Tateyamaria* sp. syn59	4.42	32	61.3	79.9	52	63.02	63.23	Seawater from the South China Sea
	Tateyamaria omphalii DOK1-4	4.31	8	61.98	77.6	54	63.18	63.2	Seawater from the East Sea in South Korea

*Jannaschia*	Jannaschia faecimaris DSM1004020	3.81	48	62.0	87.2	42	63.95	64.01	Unknown
	Jannaschia pohangensis DSM19073	3.73	18	65.5	49.5	41.3	66.88	66.79	Seashore sand in South Korea
	Jannaschia donghaensis CECT7802	3.49	67	64.6	67.2	43	66.01	66.14	Surface sea water, coast of Dokdo, South Korea

*Sulfitobacter*	*Sulfitobacter* sp. AM1-D1	4.69	6	64.9	185.2	45	65.56	65.87	Cell culture of *Alexandrium* *minutum*
	*Sulfitobacter* *noctilucicola* KCTC32123	4.09	8	57.1	107.4	50	57.38	58.15	Sea sparkle bloom region of Geoje Island in South Korea
	Sulfitobacter guttiformis KTCT32187	3.98	4	56.1	118.5	51	55.46	56.63	Costal seawater

*Roseobacter*	Roseobacter litoralis Och 149	4.75	1	57.2	93	50	58.40	59.2	Seaweed

*Oceanicola*	*Oceanicola* sp. HL-35	4.31	8	63.9	44	44	66.45	66.45	Unknown

*Shimia*	*Shimia* sp. wx04	3.78	12	58.8	54	54	60.00	60.13	Seawater from the South China Sea

*Nereida*	Nereida ignava DSM16309	2.89	36	54.0	79	47	53.52	54.38	Seawater, Mediterranean Sea

a PS, PGC superoperon.

**FIG 1:**
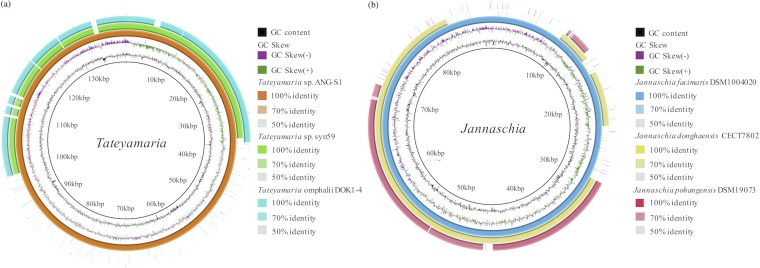
Circular maps of the PGC-containing extrachromosomal replicons for *Tateyamaria* (a) and *Jannaschia* (b) strains. (a) *Tateyamaria* sp. ANG-S1 was used as the reference. Tracks (innermost to outermost) show G+C content, GC skew (G-C/G+C), *Tateyamaria* sp. ANG-S1 synteny, *Tateyamaria* sp. syn59 synteny, and Tateyamaria omphalii DOK1-4 synteny. (b) Jannaschia faecimaris DSM 1004020 was used as the reference. Tracks (innermost to outermost) show G+C content, GC skew (G-C/G+C), Jannaschia faecimaris DSM 1004020 synteny, Jannaschia donghaensis CECT 7802 synteny, and Jannaschia pohangensis DSM19073 synteny.

*Tateyamaria* spp. have been isolated from different environments, including seawater, tidal-flat sediments, and marine animals (37–41). *Tateyamaria* spp. have also been frequently detected in algal culture bacterial communities and can occasionally dominate such communities (42–44). The *pufM* gene is present in all genomes reported for this genus (as of 28 February 2019). Three strains carrying exPGCs with similar genome sizes (average, 4.4 ± 0.8 Mb) and GC contents (average, 61.30% ± 0.70%) were chosen for analysis in this study. The sizes of the ECRs carrying exPGCs ranged from 77.6 to 139.8 kb, and the exPGCs had similar sizes (average, 53.0 ± 1.0 kb). A considerable number of highly homologous genes were present in the three PGC-containing ECRs of this genus (Fig. 1). In addition, the genes present on smaller PGC-containing ECRs were mostly also present on larger PGC-containing ECRs. Phylogenetic analysis of the replication partitioning gene (*parA*), supported by high bootstrap values among homologs, indicated that the replication modules of the three PGC-containing ECRs were highly conserved (see Fig. S1 in the supplemental material; see also Table S1 in the supplemental material).

10.1128/mSystems.00358-19.1 FIG S1 Phylogeny of the ParA plasmid partitioning proteins. The tree was constructed using maximum likelihood methods with 100 bootstrap replicates to evaluate node support. Only bootstrap values that are >50% are shown. Phototrophic strains carrying exPGCs are indicated in red. Accession numbers of all sequences are summarized in Table S1. Download FIG S1, TIF file, 2.9 MB. Copyright © 2019 Liu et al. 2019 Liu et al. This content is distributed under the terms of the Creative Commons Attribution 4.0 International license.

10.1128/mSystems.00358-19.5 TABLE S1 Accession numbers of partitioning replication gene *parA* and 16S rRNA genes and whole-genome sequences of 38 AAPB strains used for phylogenetic analyses. Download Table S1, DOCX file, 0.02 MB. Copyright © 2019 Liu et al. 2019 Liu et al. This content is distributed under the terms of the Creative Commons Attribution 4.0 International license.

*Jannaschia* is an ecologically important genus of AAPB (45). Indeed, 25% to 30% of all AAPB 16S rRNA gene clone sequences from samples collected in the central Baltic Sea belonged to *Jannaschia-*related bacteria (46). Among known *Jannaschia* isolates, CCS1 is the only strain observed to conduct photoheterotrophy (47, 48). Twelve *Jannaschia* strains were available for analysis with whole-genome sequences, with six containing PGCs; three of the six were exPGC types, and the other three were cPGC types. The PGC-containing ECRs ranged in size from 49.5 to 87.2 kb, while their exPGCs were ∼45 kb in length. The sizes of the genomes of the three *Jannaschia* strains carrying exPGCs ranged from 3.49 to 3.81 Mb, with GC contents ranging from 62.0% to 65.5%. The three PGC-containing ECRs of *Jannaschia* shared a large syntenic region comprising ∼50 kb, and the larger PGC-containing ECRs contained genes that were carried by the PGC-containing smaller ECRs, as observed for the ECRs in the *Tateyamaria* genomes (Fig. 1). In addition, their replicon replication modules were closely related based on a phylogenetic analysis of *parA* genes (Fig. S1; see also Table S1).

*Sulfitobacter* spp. are widely distributed in different marine environments and may play important roles in organic sulfur cycling (49–54). Culture-independent surveys of AAPB have indicated that *Sulfitobacter* spp. account for a significant fraction of AAPB communities in natural environments (10, 55, 56). However, only a few *Sulfitobacter* AAPB strains have been isolated and described (10, 51, 57). Genomes have been sequenced from only three *Sulfitobacter* AAPB strains, and the data revealed that their PGCs were located on ECRs. The three genomes ranged in size from 3.98 to 4.69 Mb, with GC contents ranging from 56.1% to 64.9%. The three PGC-containing ECRs exhibited sizes greater than 100 kb, while the three exPGCs were 45, 50, and 51 kb. Other than genes involved in photosynthesis, only a small number of genes were shared by PGC-containing ECRs in *Sulfitobacter*, which contrasted with the high degree of conservation observed for ECRs of *Tateyamaria* and *Jannaschia*. Furthermore, the *parA* genes within the three ECRs of *Sulfitobacter* were phylogenetically very distinct (Fig. S1; see also Table S1).

Roseobacter litoralis Och 149 was the first AAPB bacterium described (in 1991) and contained a linear ECR harboring a PGC (58, 59). The genus comprises only two species, R. litoralis Och 149 and *R. denitrificans* OCh114, with the latter containing a cPGC (58). In contrast, little is known about phototrophic species within *Oceanicola*, *Shimia*, and *Nereida*. *Oceanicola* sp. HL-35 exhibits 99% 16S rRNA gene sequence identity with Lacimonas salitoletrans TS-T30, which lacks a PGC (60). Prior to the isolation of *Shimia* sp. wx04, *Shimia* spp. were thought to be strict chemoorganotrophs based on culture-dependent investigations (61–65). Finally, Nereida ignava DSM 16093 was isolated from waters of the Mediterranean and is the sole species currently described for the *Nereida* genus (66, 67).

### Identification of PGC-containing chromid-like ECRs and PGC-containing plasmid-like ECRs.

**FIG 2:**
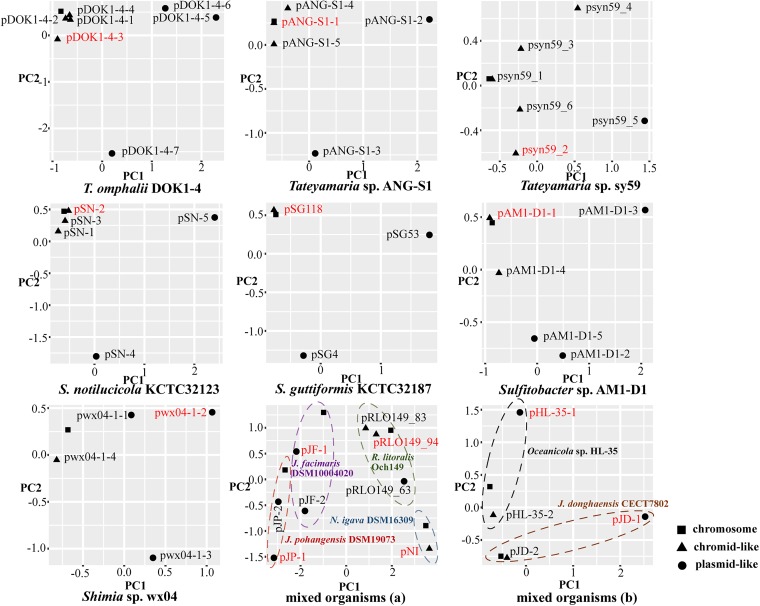
Principal-component analysis of the relative synonymous codon usage (RSCU) of replicons from the 13 strains carrying exPGCs. Chromsomes, chromids, and plasmids are indicated by squares, triangles, and circles, respectively. The strains containing more than three replicons were analyzed on a strain-by-strain basis, while the rest were mixed and then analyzed. The replicons containing PGCs are indicated in red.

Chromids represent a novel type of ECRs that were recently described as genetic elements that are distinct from both chromosomes and plasmids (68). Since chromids typically carry essential genes, they remain more stably present than plasmids in bacteria and are considered indispensable for bacterial hosts (68). Comparison of the relative synonymous codon usage (RSCU) levels of bacterial chromosomes and ECRs can help identify if ECRs are chromids or not, as RSCU levels of chromid are similar to those of the corresponding chromosomes (34, 68, 69). Principal-component analysis (PCA) of the RSCU levels of all replicons from the 13 strains were analyzed for use in classifying elements as chromids and plasmids. The analyses indicated that eight PGC-containing ECRs (*Tateyamaria* sp. ANG-S1, *Tateyamaria* sp. syn59, *T. omphalii* DOK1-4, *Sulfitobacter* sp. AM1-D1, Sulfitobacter noctilucicola KCTC 32123, S. guttiformis KTCT 32187, R. litoralis Och 149, and N. ignava DSM16309) could be clearly assigned as chromid-like ECRs, while the other ECRs containing PGCs were provisionally classified as plasmid-like ECRs (Fig. 2; see also Table S2).

10.1128/mSystems.00358-19.6 TABLE S2 The extrachromosomal replicon types for 13 strains. Download Table S2, DOCX file, 0.01 MB. Copyright © 2019 Liu et al. 2019 Liu et al. This content is distributed under the terms of the Creative Commons Attribution 4.0 International license.

### Phylogenetic analysis.

A phylogenetic analysis based on 16S rRNA gene nucleotide sequences from 13 exPGC-containing bacterial strains and 43 reference strains was conducted with photoheterotrophs and heterotrophs to show the phylogenetic distribution of the 13 strains carrying exPGCs (Fig. S2). As observed for the cPGC-containing AAPB, the 13 exPGC-containing bacterial strains did not comprise a monophyletic phylogenetic group but were instead distributed throughout the 16S rRNA phylogenetic tree.

10.1128/mSystems.00358-19.2 FIG S2 16S rRNA gene sequence phylogenetic analysis indicating the patchy distribution of AAPB within the *Roseobacter* clade. The tree was constructed using maximum likelihood methods with 100 bootstrap replicates to evaluate node support. Only bootstrap values that are >50% are shown. Phototrophic strains are indicated by bolding, and those carrying exPGCs are indicated in red. Accession numbers of all sequences are summarized in Table S1. Download FIG S2, TIF file, 2.6 MB. Copyright © 2019 Liu et al. 2019 Liu et al. This content is distributed under the terms of the Creative Commons Attribution 4.0 International license.

**FIG 3:**
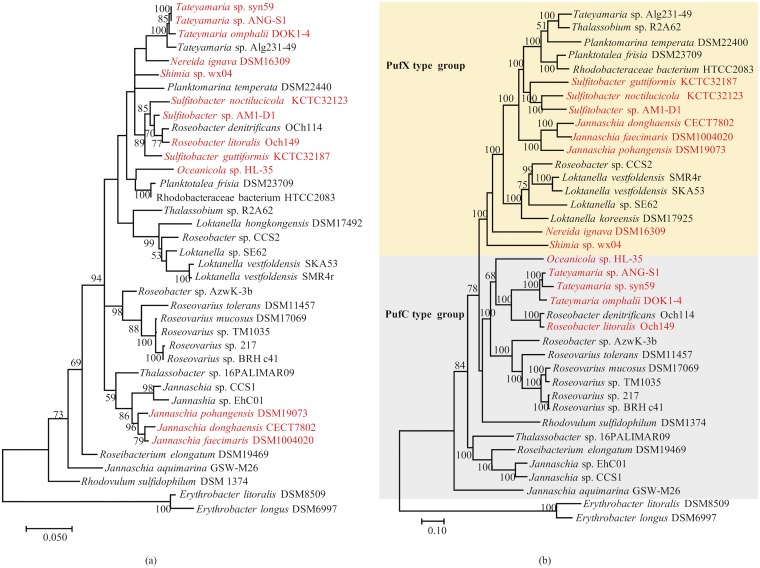
Phylogenetic trees of 16S rRNA genes (a) and concatenation of 29 conserved PGC genes (b). Trees were constructed using maximum likelihood methods with 100 bootstrap replicates to evaluate node support. Only bootstrap values that were >50% are shown. Phototrophic strains carrying exPGCs are indicated in red. Accession numbers of all sequences are summarized in Table S1.

To further investigate the phylogenetic relationships of the *Roseobacter* clade strains, 38 photoheterotrophic bacterial genomes were subjected to phylogenetic analysis using 29 conserved PGC genes. Comparison of phylogenies based on 16S rRNA nucleotide sequences and amino acid sequences of 29 conserved genes of the PGC revealed considerable topological differences (Fig. 3). For example, the four *Tateyamaria* strains clustered and were shown to be closely related to the N. ignava DSM16309 strain in the 16S rRNA gene phylogenetic analysis. However, the *Tateyamaria* strains were using the 29 highly conserved genes of the PGC, with *Tateyamaria* sp. Alg231-49 closely related to *Thalassonium* sp. R2A62 and the other three strains associated with the *Roseobacter* species. Similarly, the five *Jannaschia* strains formed a group with (86%) bootstrap support based on the 16S rRNA phylogenetic analysis; however, they were clearly differentiated into two distant subtrees in the PGC-based phylogenetic analysis.

In addition, the PGC-based phylogenetic analysis indicated the presence of two phylogenetic groups corresponding to differences in their *puf* operon structures. Specifically, the groups corresponded to PufC-containing and PufX-containing groups (Fig. 3b). In particular, *Tateyamaria* sp. syn59, T. omphalli pDOK1-4, *Tateyamaria* sp. ANG-S1, R. litoralis Och149, and *Oceanicola* sp. HL-35 contained *pufC* genes, while the others contained *pufX* genes. Five of the exPGCs in the PufC group clustered together, suggesting common ancestry for these exPGCs. Moreover, the cPGC in Roseobacter denitrificans Och114 represented a basal clade to a subtree also comprising the five aforementioned exPGCs, thereby providing evidence for chromosomal reintegration from an ECR (33). Furthermore, the externally nested position of the exPGC from *Oceanicola* sp. HL-35 within this subtree indicated that the exPGC was possibly transferred from other AAPB strains and could be further transferred to other distant bacterial strains via an ECR. Nine of the 13 exPGCs belonged to three genera among the seven that were analyzed. Among these, the exPGCs from strains of the same genus were closely related phylogenetically, suggesting that the transfer of exPGCs was more likely to occur among strains within the same genus. Two types of *puf* operon structures were observed within the genomes of different strains within the *Tateyamaria* and *Jannaschia* genera. Among the six PGC-containing *Jannaschia* strains, three exPGCs were PufX types whereas the other three were PufC types. Similarly, among the four phototrophic *Tateyamaria* strains analyzed, one cPGC was a PufX type whereas the other three exPGCs were PufC types.

### exPGC structures and arrangements.

**FIG 4:**
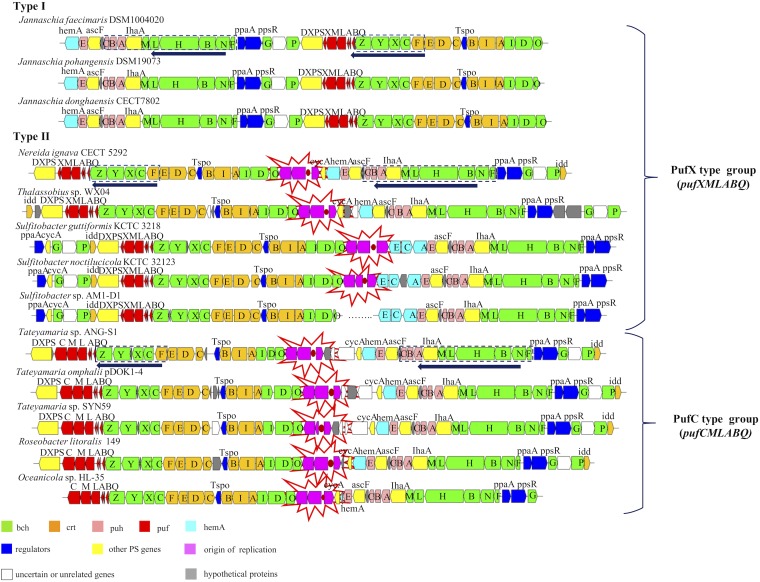
Photosynthetic gene cluster structures and arrangements for 13 exPGCs. ECR module positions (plasmid origin replication) within the exPGC are shown in red and highlighted by stars. Arrows indicate the order of the conserved PGC genes as follows: type I, forward *puh-LhaA-bchMLHNF* plus forward *puf-bchZYXC-crtF*; type II, forward *puf-bchZYXC-crtF* plus forward *puh-LhaA-bchMLHNF*. The classifications of type PufX and type PufC groups are based on their different *puf* operon compositions, corresponding to *pufXMLABQ* and *pufCMLABQ*.

Three different structures were observed among the 13 exPGCs (Fig. 4). The 41 photosynthetic genes on the ECR of the three *Jannaschia* strains were organized into one superoperon with the same structure as that of the cPGC (20). In addition, the exPGC of *Sulfitobacter* sp. AM1-D1 was separated by more than 100 genes between *bchIDO* and *hemECA*. The other exPGCs all appeared to have been inserted by their replication modules, as previously identified (31, 33).

The arrangement of photosynthetic genes is also a key characteristic of PGCs in AAPB. Three forms of PGC arrangement have been observed in *Roseobacter* clade organisms based on a combination of two conserved regions (*puh-LhaA-bchMLHNF* and *puf-bchZYXC-crtF*) (20). The 13 exPGCs contained the same conserved gene order as the cPGCs and comprised two different types. The type I arrangement was exhibited by exPGCs of the three *Jannaschia* strains (J. pohangensis DSM19073, J. donghaensis CECT7802, and J. faecimaris DSM10004020), wherein genes were arranged as forward *puh LhaA bchMLHNF* plus forward *puf-bchZYXC-crtF*. Type II arrangements were observed for the other 10 strains, wherein arrangements followed the pattern of forward *puf-bchZYXC-crtF* plus forward *puh*-*LhaA-bchMLHNF* (Fig. 4).

The arrangements of exPGCs of the PufC-containing group were of type II, which exhibited the high conservation in the direction and order of all photosynthetic genes on the exPGCs. In contrast, the exPGCs in the PufX-containing group exhibited two different types of arrangements, with unique traits present in different genera. For example, *hemC* and *hemE* genes encoding tetrapyrrole biosynthesis proteins were present only in the exPGCs of *Sulfitobacter*. In addition, the genomic region ranging from *bchG* to *idd* was located upstream of the *puf* operon in *Sulfitobacter*, although it is typically downstream of the *ppaA* and *ppsR* regulator genes within PGCs (20). Furthermore, the three exPGCs of the *Jannaschia* strains lacked cytochrome *c*_2_ (*cyc2*) and diphosphate delta-isomerase (*idi*) genes. *cyc2* is involved in electron transfer, while *idi* is involved in isoprenoid biosynthesis. The loss of these genes is not lethal for phototrophic bacteria (70, 71), but the genes are nevertheless expected to be present in the PGCs of phototrophic bacteria within the *Roseobacter* clade (33, 72, 73).

### Evidence of ECR-mediated PGC transfer within the *Roseobacter* clade.

Recent studies have suggested that ECRs could be vehicles for HGT of PGCs (31, 33), albeit with limited evidence. The idea of transfer of PGCs by ECRs was supported in our analyses by the coexistence of two different types of *puf* operon structures (PufC and PufX types) in different strains of two genera, *Tateyamaria* and *Jannaschia.* In particular, these two types of *puf* operons were located on cPGCs and exPGCs, respectively. The Global Ocean Sampling expedition metageomes were the first to reveal that *pufC* could be replaced by *pufX* in AAPB and that *pufC* and *pufX* were present in different AAPB phylogroups (5, 74). Thus, phylogenetic divergence of the two types of *puf* operons in strains from the same genus suggested that one or both of them were introduced by other phototrophic phylogroups. Moreover, phylogenetic congruence between whole PGCs and conserved photosynthetic operons within the PGC (i.e., *bchFNBHLM-IhaA-puhABC* and *pufMLABQ-bchZYXC-crtF*) indicate that PGCs act as entire functional units rather than being subject to partial transfer between strains (Fig. S3); this is consistent with a previous study (33). ECRs are mobile genetic elements; thus, PGCs carried by ECRs are more likely to be horizontally transferred.

10.1128/mSystems.00358-19.3 FIG S3 Phylogenetic tree of the two conserved PGC subclusters based on concatenated bchFNBHLM-IhaA-puhABC (a) and pufMLAB-bchZYXC-crtF (b) protein sequences. The tree was constructed using maximum likelihood methods with 100 bootstrap replicates to evaluate node support. Only bootstrap values that are >50% are shown. Phototrophic strains carrying exPGCs are indicated in red. Accession numbers of all sequences are summarized in Table S1. Download FIG S3, TIF file, 2.5 MB. Copyright © 2019 Liu et al. 2019 Liu et al. This content is distributed under the terms of the Creative Commons Attribution 4.0 International license.

### The potential for transfer of PGC-containing ECRs.

As described above, the 13 PGC-containing ECRs were divided into two types based on their sizes and functions. Small PGC-containing ECRs within *Oceanicola* sp. HL-35, *Shimia* sp. wx04, and J. pohangensis DSM19073 carried more than 80% of the genes coding for PGCs. These ECRs are usually present as plasmids and are likely to play an important role in the transfer of phototrophic capacities among species. This is especially probable because the transfer of small plasmids achieves higher efficiencies and the three streamlined PGC-containing ECRs still appear to confer the capability of chlorophototrophy (75, 76). The acquisition of streamlined PGC-containing ECRs might enable strains to obtain new lifestyles at low costs, thereby providing advantages under certain environmental conditions (34). The other large PGC-containing ECRs also encoded proteins with various nonphotosynthetic functions. Moreover, a *sox* gene cluster (*soxRSVYAZBCD*), usually located on the bacterial chromosome, was observed on the PGC-containing chromid-like elements of N. ignava DSM1630, suggesting that the *sox* gene cluster might be also transferred by the ECR. Most of these large ECRs were classified as chromid-like ECRs. Consequently, these PGC-containing ECRs might preferentially be maintained in bacterial hosts rather than be transferred among hosts. Notably, PGCs carried by both plasmid-like and chromid-like ECRs have been suggested to be genomically stable because most exPGCs have been inserted by their corresponding ECR replication modules (31).

Comparison of the GC contents of the bacterial genomes, exPGCs, and PGC-containing ECRs did not reveal significant differences for any of the 13 *Roseobacter* clade strains (Fig. S4). Thus, the transfer of these PGC-containing ECRs into bacteria likely occurred during very distant evolutionary events or, otherwise, only between closely related species (77).

10.1128/mSystems.00358-19.4 FIG S4 GC content correlations among 13 strains of *Roseobacter* species for genomic GC content and that of PGC-containing ECRs (a), genomes and exPGCs (b), and PGC-containing ECRs and exPGCs (c). Download FIG S4, TIF file, 0.3 MB. Copyright © 2019 Liu et al. 2019 Liu et al. This content is distributed under the terms of the Creative Commons Attribution 4.0 International license.

### A scenario to explain the evolution of AAPB exPGCs in the *Roseobacter* clade.

**FIG 5:**
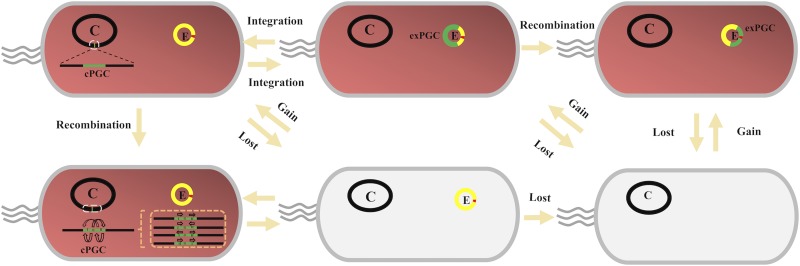
Scenarios to explain the transfer and evolution of exPGCs within *Roseobacter* clade species. PGCs are indicated in green, and PGC-containing ECR origins of replication are indicated in red. Photosynthetic bacteria are indicated in light red, while non-photosynthetic bacteria are indicated in light gray. C, chromosome; E, PGC-containing ECR.

A previous scenario was suggested to explain the evolution of exPGCs in *Roseobacter* clade organisms, wherein a chromosomal PGC superoperon was transferred into an ECR, followed by integration of replication origin genes into the ECR (31, 34). Our analyses validate this explanation, and we further present a more detailed scenario (Fig. 5) to explain the transfer of PGCs within the *Roseobacter* clade after analyzing genomic and evolutionary characteristics of 13 exPGCs in this group (31). In this revised scenario, PGCs were first initially translocated from chromosomes to ECRs, as represented by the superoperon structure of exPGCs from J. faecimaris DSM1004020, J. pohangensis DSM19073, and J. donghaensis CECT 7802. Three subsequent transfer possibilities are present for exPGCs: (i) exPGCs reintegrated into chromosomes and became cPGCs, as observed for most phototrophic *Roseobacter* clade strains; (ii) PGC-containing ECRs were lost from strains, such that the bacteria became heterotrophs; or (iii) exPGCs carried by ECRs were subjected to further recombination and became stable within ECRs. A remarkable characteristic of the majority of exPGCs is the insertion of an ECR replication module within the PGC as a result of a series of recombination events. Such an event could have helped ensure the stability of exPGCs (31).

The patchy distribution of AAPB within the *Roseobacter* clade has been explained by two evolutionary models that invoke either loss or gain of PGCs (11–13). Given that ECRs have played a critical role in the loss or gain of PGCs during the evolutionary history of photosynthesis, the patchy distribution of AAPB within the *Roseobacter* clade can be plausibly explained by ECR-mediated mechanisms. To date, exPGCs have accounted for ∼20% of all PGCs in the currently available genomes of *Roseobacter* clade strains (Table S3), highlighting their prevalence in these organisms. It is likely that additional exPGCs carried by other strains will be identified with further generation of new bacterial genome sequences. Moreover, we suggest that the gain and loss of PGCs, as mediated by chromosomes and especially ECRs, resulted in the patchy distribution of AAPB within the *Roseobacter* clade.

10.1128/mSystems.00358-19.7 TABLE S3 Whole-genome sequences for 67 AAPB strains within the *Roseobacter* clade that are publicly available in NCBI. AAPB strains carrying exPGCs are indicated in red. Download Table S3, DOCX file, 0.01 MB. Copyright © 2019 Liu et al. 2019 Liu et al. This content is distributed under the terms of the Creative Commons Attribution 4.0 International license.

In the present study, the genomic characteristics and evolution of 13 PGCs carried by ECRs were analyzed. The coexistence of two types of *puf* operon structures within strains of the same genera provided clear evidence of the horizontal transfer of PGCs mediated by ECR. Analysis of PGC-containing plasmid-like and chromid-like ECRs indicated that exPGCs could stably exist in bacteria after transfer, highlighting the importance of phototrophic metabolism carried by ECRs for some bacteria. Furthermore, these analyses indicated that the process of gain or loss of PGCs, as mediated by ECRs, contributes to the patchy distribution of phototrophic capacities within the *Roseobacter* clade.

## MATERIALS AND METHODS

### Strain isolation.

*Tateyamaria* sp. syn59 and *Shimia* sp. wx04 were isolated from the South China Sea in April 2016 using oligotrophic medium F/2 plates (78), followed by transfer onto rich organic liquid medium (Marine Broth 2216; Difco, USA) for further isolation and cultivation. All cultures were incubated at 28°C with shaking at 200 rpm in the dark. Genomic DNA from the two strains was extracted using a TaKaRa MiniBEST universal genomic DNA extraction kit (Japan).

### Genome sequencing, assembly, and annotation.

The genomes of *Tateyamaria* sp. syn59 and *Shimia* sp. wx04 were sequenced on an Illumina MiSeq platform (Illumina, USA). Specifically, 2 × 250-bp paired-end read sequencing was conducted, followed by read assembly using the Velvet program (version 2.8) (79). Prediction and annotation of open reading frames (ORFs) were conducted using a Rapid Annotation using Subsystems Technology (RAST) platform. Further, annotations of plasmids and exPGCs were validated by BLASTP searches against the National Center for Biotechnology Information (NCBI) nonredundant (nr) protein database (https://blast.ncbi.nlm.nih.gov/Blast.cgi). The whole-genome sequences of *Tateyamaria* sp. syn59 and *Shimia* sp. wx04 are available under GenBank accession numbers VCBA00000000.1 (https://www.ncbi.nlm.nih.gov/nuccore/VCDK00000000) and VCDK00000000.1 (https://www.ncbi.nlm.nih.gov/nuccore/VCBA00000000), respectively.

### Retrieval of AAPB genomes from GenBank.

Genome sequence data for the other eleven *Roseobacter* clade strains were obtained from NCBI, including data for Tateyamaria omphalii DOK1-4 (CP019312.1); *Tateyamaria* sp. ANG-S1 (JWLL00000000.1); Roseobacter litoralis Och 149 (CP002623.1); *Oceanicola* sp. HL-35 (JAFT00000000.1); Nereida ignava DSM 16309 (CVPC00000000.1); Sulfitobacter noctilucicola KCTC 32123 (JASD00000000.1); Sulfitobacter guttiformis KCTC 32187 (JASG00000000.1); *Sulfitobacter* sp. AM1-D1 (CP018076.1); Jannaschia donghaensis CECT 7802 (NZ_CXSU00000000.1); Jannaschia faecimaris DSM 10004020 (FNPX00000000.1); and Jannaschia pohangensis DSM 19073 (FORA00000000.1).

### Phylogenetic analysis.

Complete 16S rRNA gene sequences were extracted from the whole-genome assemblies (WGA) using the Cluster program (80). To construct a phylogeny for the PGCs, the amino acid sequences of 29 conserved photosynthetic genes within PGCs (*bchI*, *bchD*, *bchO*, *tspO*, *crtC*, *crtD*, *crtF*, *bchC*, *bchX*, *bchY*, *bchZ*, *pufL*, *pufM*, *bchP*, *puCC*, *bchG*, *ppsR*, *bchF*, *bchN*, *bchB*, *bchH*, *bchL*, *bchM*, *IhaA*, *puhA*, *puhB*, *puhC*, *ascF*, and *puhE*) (see Table S4 in the supplemental material) were retrieved from the query genomes and then individually aligned using ClustalW, as implemented in the BioEdit program (81). Phylogenetic analyses were conducted using RAxML 8.02 (82) and maximum likelihood (ML) methods. The robustness of the tree topologies was evaluated using bootstrap analysis with 100 replicates. Final trees were then visualized using the Interactive Tree of Life viewer and MEGA version 7.0 (83, 84).

10.1128/mSystems.00358-19.8 TABLE S4 The list of 29 conserved genes in the PGC. Download Table S4, DOCX file, 0.01 MB. Copyright © 2019 Liu et al. 2019 Liu et al. This content is distributed under the terms of the Creative Commons Attribution 4.0 International license.

### Data availability.

All data used in this study are publicly available in GenBank. Accession numbers can be found in Table S1 and Table S3.

## References

[1] Jiao N, Zhang Y, Zeng Y, Hong N, Liu R, Chen F, Wang P 2007 Distinct distribution pattern of abundance and diversity of aerobic anoxygenic phototrophic bacteria in the global ocean. Environ Microbiol 9:3091–3099. doi:10.1111/j.1462-2920.2007.01419.x. 17991036

[2] Ritchie AE, Johnson ZI 2012 Abundance and genetic diversity of aerobic anoxygenic phototrophic bacteria of coastal regions of the Pacific Ocean. Appl Environ Microbiol 78:2858–2866. doi:10.1128/AEM.06268-11. 22307290 PMC3318826

[3] Koblížek M 2015 Ecology of aerobic anoxygenic phototrophs in aquatic environments. FEMS Microbiol Rev 39:854–870. doi:10.1093/femsre/fuv032. 26139241

[4] Jiao N, Zhang F, Hong N 2010 Significant roles of bacteriochlorophylla supplemental to chlorophylla in the ocean. ISME J 4:595–597. doi:10.1038/ismej.2009.135. 20010633

[5] Yutin N, Suzuki MT, Teeling H, Weber M, Venter JC, Rusch DB, Béjà O 2007 Assessing diversity and biogeography of aerobic anoxygenic phototrophic bacteria in surface waters of the Atlantic and Pacific Oceans using the Global Ocean Sampling expedition metagenomes. Environ Microbiol 9:1464–1475. doi:10.1111/j.1462-2920.2007.01265.x. 17504484

[6] Kolber ZS, Plumley FG, Lang AS, Beatty JT, Blankenship RE, VanDover CL, Vetriani C, Koblizek M, Rathgeber C, Falkowski PG 2001 Contribution of aerobic photoheterotrophic bacteria to the carbon cycle in the ocean. Science 292:2492–2495. doi:10.1126/science.1059707. 11431568

[7] Koblížek M, Masín M, Ras J, Poulton AJ, Prášil O 2007 Rapid growth rates of aerobic anoxygenic phototrophs in the ocean. Environ Microbiol 9:2401–2406. doi:10.1111/j.1462-2920.2007.01354.x. 17803766

[8] Lami R, Cottrell MT, Ras J, Ulloa O, Obernosterer I, Claustre H, Kirchman DL, Lebaron P 2007 High abundances of aerobic anoxygenic photosynthetic bacteria in the South Pacific Ocean. Appl Environ Microbiol 73:4198–4205. doi:10.1128/AEM.02652-06. 17496136 PMC1932784

[9] Cottrell MT, Mannino A, Kirchman DL 2006 Aerobic anoxygenic phototrophic bacteria in the Mid-Atlantic Bight and the North Pacific Gyre. Appl Environ Microbiol 72:557–564. doi:10.1128/AEM.72.1.557-564.2006. 16391092 PMC1352302

[10] Boeuf D, Cottrell MT, Kirchman DL, Lebaron P, Jeanthon C 2013 Summer community structure of aerobic anoxygenic phototrophic bacteria in the western Arctic Ocean. FEMS Microbiol Ecol 85:417–432. doi:10.1111/1574-6941.12130. 23560623

[11] Yurkov V, Hughes E 2017 Aerobic anoxygenic phototrophs: four decades of mystery, p 193–214. In Modern Topics in the Phototrophic Prokaryotes. Springer, Cham.

[12] Beatty JT 2002 On the natural selection and evolution of the aerobic phototrophic bacteria. Photosynth Res 73:109–114. doi:10.1023/A:1020493518379. 16245110

[13] Yurkov V, Csotonyi JT 2009 New light on aerobic anoxygenic phototrophs, p 31–55. *In* The purple phototrophic bacteria, 3rd ed Springer, Dordrecht, Netherlands.

[14] Bekker A, Holland HD, Wang PL, Rumble D III, Stein HJ, Hannah JL, Coetzee LL, Beukes NJ 2004 Dating the rise of atmospheric oxygen. Nature 427:117. doi:10.1038/nature02260. 14712267

[15] Holland HD 2006 The oxygenation of the atmosphere and oceans. Philos Trans R Soc B 361:903–915. doi:10.1098/rstb.2006.1838. PMC1578726 16754606

[16] Shimada K 1995 Aerobic anoxygenic phototrophs, p 105–122. *In* Anoxygenic photosynthetic bacteria. Kluwer Academic Publishers, Dordrecht, Netherlands.

[17] Yurkov VV, Beatty JT 1998 Aerobic anoxygenic phototrophic bacteria. Microbiol Mol Biol Rev 62:695–724. 9729607 10.1128/mmbr.62.3.695-724.1998 PMC98932

[18] Nishimura K, Shimada H, Ohta H, Masuda T, Shioi Y, Takamiya K-I 1996 Expression of the puf operon in an aerobic photosynthetic bacterium, Roseobacter denitrificans. Plant Cell Physiol 37:153–159. doi:10.1093/oxfordjournals.pcp.a028926. 8665093

[19] Beatty JT 1995 Organization of photosynthesis gene transcripts, p 1209–1219. *In* Anoxygenic photosynthetic bacteria. Springer, Dordrecht, Netherlands.

[20] Zheng Q, Zhang R, Koblížek M, Boldareva EN, Yurkov V, Yan S, Jiao N 2011 Diverse arrangement of photosynthetic gene clusters in aerobic anoxygenic phototrophic bacteria. PLoS One 6:e25050. doi:10.1371/journal.pone.0025050. 21949847 PMC3176799

[21] Liotenberg S, Steunou AS, Picaud M, Husson FR, Astier C, Ouchane S 2008 Organization and expression of photosynthesis genes and operons in anoxygenic photosynthetic proteobacteria. Environ Microbiol 10:2267–2276. doi:10.1111/j.1462-2920.2008.01649.x. 18479441

[22] Swingley WD, Blankenship RE, Raymond J 2009 Evolutionary relationships among purple photosynthetic bacteria and the origin of proteobacterial photosynthetic systems, p 17–29. *In* The purple phototrophic bacteria. Springer, Dordrecht, Netherlands.

[23] Zheng Q, Lin W, Liu Y, Chen C, Jiao N 2016 A comparison of 14 Erythrobacter genomes provides insights into the genomic divergence and scattered distribution of phototrophs. Front Microbiol 7:775. 27446024 10.3389/fmicb.2016.00984 PMC4919336

[24] Béjà O, Suzuki MT, Heidelberg JF, Nelson WC, Preston CM, Hamada T, Eisen JA, Fraser CM, DeLong EF 2002 Unsuspected diversity among marine aerobic anoxygenic phototrophs. Nature 415:630–633. doi:10.1038/415630a. 11832943

[25] Zeng Y, Feng F, Medová H, Dean J, Koblížek M 2014 Functional type 2 photosynthetic reaction centers found in the rare bacterial phylum Gemmatimonadetes. Proc Natl Acad Sci U S A 111:7795–7800. doi:10.1073/pnas.1400295111. 24821787 PMC4040607

[26] Zheng Q, Zhang R, Fogg PCM, Beatty JT, Wang Y, Jiao N 2012 Gain and loss of phototrophic genes revealed by comparison of two Citromicrobium bacterial genomes. PLoS One 7:e35790. doi:10.1371/journal.pone.0035790. 22558224 PMC3338782

[27] Newton RJ, Griffin LE, Bowles KM, Meile C, Gifford S, Givens CE, Howard EC, King E, Oakley CA, Reisch CR, Rinta-Kanto JM, Sharma S, Sun S, Varaljay V, Vila-Costa M, Westrich JR, Moran MA 2010 Genome characteristics of a generalist marine bacterial lineage. ISME J 4:784–798. doi:10.1038/ismej.2009.150. 20072162

[28] Luo H, Moran MA 2014 Evolutionary ecology of the marine Roseobacter clade. Microbiol Mol Biol Rev 78:573–587. doi:10.1128/MMBR.00020-14. 25428935 PMC4248658

[29] Brinkhoff T, Giebel H-A, Simon M 2008 Diversity, ecology, and genomics of the Roseobacter clade: a short overview. Arch Microbiol 189:531–539. doi:10.1007/s00203-008-0353-y. 18253713

[30] Nagashima KVP, Hiraishi A, Shimada K, Matsuura K 1997 Horizontal transfer of genes coding for the photosynthetic reaction centers of purple bacteria. J Mol Evol 45:131–136. doi:10.1007/PL00006212. 9236272

[31] Petersen J, Brinkmann H, Bunk B, Michael V, Päuker O, Pradella S 2012 Think pink: photosynthesis, plasmids and the Roseobacter clade. Environ Microbiol 14:2661–2672. doi:10.1111/j.1462-2920.2012.02806.x. 22732061

[32] Raymond J, Zhaxybayeva O, Gogarten JP, Gerdes SY, Blankenship RE 2002 Whole-genome analysis of photosynthetic prokaryotes. Science 298:1616–1620. doi:10.1126/science.1075558. 12446909

[33] Brinkmann H, Göker M, Koblížek M, Wagner-Döbler I, Petersen J 2018 Horizontal operon transfer, plasmids, and the evolution of photosynthesis in Rhodobacteraceae. ISME J 12:1994–2010. doi:10.1038/s41396-018-0150-9. 29795276 PMC6052148

[34] Petersen J, Frank O, Göker M, Pradella S 2013 Extrachromosomal, extraordinary and essential–the plasmids of the Roseobacter clade. Appl Microbiol Biotechnol 97:2805–2815. doi:10.1007/s00253-013-4746-8. 23435940

[35] Sørensen SJ, Bailey M, Hansen LH, Kroer N, Wuertz S 2005 Studying plasmid horizontal transfer in situ: a critical review. Nat Rev Microbiol 3:700–710. doi:10.1038/nrmicro1232. 16138098

[36] Thomas CM, Nielsen KM 2005 Mechanisms of, and barriers to, horizontal gene transfer between bacteria. Nat Rev Microbiol 3:711–721. doi:10.1038/nrmicro1234. 16138099

[37] Kurahashi M, Yokota A 2007 Tateyamaria omphalii gen. nov., sp. nov., an α-Proteobacterium isolated from a top shell Omphalius pfeifferi pfeifferi. Syst Appl Microbiol 30:371–375. doi:10.1016/j.syapm.2006.11.007. 17207953

[38] Sass H, Köpke B, Rutters H, Feuerlein T, Droge S, Cypionka H, Engelen B 2010 Tateyamaria pelophila sp. nov., a facultatively anaerobic alphaproteobacterium isolated from tidal-flat sediment, and emended descriptions of the genus Tateyamaria and of Tateyamaria omphalii. Int J Syst Evol Microbiol 60:1770–1777. doi:10.1099/ijs.0.013524-0. 19749035

[39] Jeanthon C, Boeuf D, Dahan O, Gall FL, Garczarek L, Bendif EM, Lehours AC 2011 Diversity of cultivated and metabolically active aerobic anoxygenic phototrophic bacteria along an oligotrophic gradient in the Mediterranean Sea. Biogeosciences 8:1955–1970. doi:10.5194/bg-8-1955-2011.

[40] Collins AJ, Fullmer MS, Gogarten JP, Nyholm SV 2015 Comparative genomics of Roseobacter clade bacteria isolated from the accessory nidamental gland of Euprymna scolopes. Front Microbiol 6:11524. 10.3389/fmicb.2015.00123 PMC4337385 25755651

[41] Karimi E 2018 Metagenomics and functional genomics of bacterial symbionts of Spongia (Porifera, Dictyoceratida) specimens from the Algarvian shore (South Portugal). PhD thesis University of Algarve, Faro, Portugal.

[42] Huggett MJ, McMahon K, Bernasconi R 2018 Future warming and acidification result in multiple ecological impacts to a temperate coralline alga. Environ Microbiol 20:2769–2782. doi:10.1111/1462-2920.14113. 29575500

[43] Bengtsson MM, Sjøtun K, Lanzén A, Øvreås L 2012 Bacterial diversity in relation to secondary production and succession on surfaces of the kelp Laminaria hyperborea. ISME J 6:2188–2198. doi:10.1038/ismej.2012.67. 22763650 PMC3505018

[44] Kalitnik AA, Nedashkovskaya OI, Stenkova AM, Yermak IM, Kukhlevskiy AD 2018 Carrageenanolytic enzymes from marine bacteria associated with the red alga Tichocarpus crinitus. J Appl Phycol 30:2071–2081. doi:10.1007/s10811-017-1355-4.

[45] Zeng YH, Chen XH, Jiao NZ 2007 Genetic diversity assessment of anoxygenic photosynthetic bacteria by distance-based grouping analysis of pufM sequences. Lett Appl Microbiol 45:639–645. doi:10.1111/j.1472-765X.2007.02247.x. 17922815

[46] Salka I, Moulisová V, Koblížek M, Jost G, Jürgens K, Labrenz M 2008 Abundance, depth distribution, and composition of aerobic bacteriochlorophyll a-producing bacteria in four basins of the central Baltic Sea. Appl Environ Microbiol 74:4398–4404. doi:10.1128/AEM.02447-07. 18502937 PMC2493182

[47] Moran MA, Belas R, Schell MA, González JM, Sun F, Sun S, Binder BJ, Edmonds J, Ye W, Orcutt B, Howard EC, Meile C, Palefsky W, Goesmann A, Ren Q, Paulsen I, Ulrich LE, Thompson LS, Saunders E, Buchan A 2007 Ecological genomics of marine Roseobacters. Appl Environ Microbiol 73:4559–4569. doi:10.1128/AEM.02580-06. 17526795 PMC1932822

[48] Pujalte MJ, Lucena T, Ruvira MA, Arahal DR, Macián MC 2014 The family rhodobacteraceae, p 439–512. *In* The prokaryotes. Springer, Berlin, Germany.

[49] Prabagaran SR, Manorama R, Delille D, Shivaji S 2007 Predominance of Roseobacter, Sulfitobacter, Glaciecola and Psychrobacter in seawater collected off Ushuaia, Argentina, sub-Antarctica. FEMS Microbiol Ecol 59:342–355. doi:10.1111/j.1574-6941.2006.00213.x. 17026513

[50] Rooney-Varga JN, Giewat MW, Savin MC, Sood S, LeGresley M, Martin JL 2005 Links between phytoplankton and bacterial community dynamics in a coastal marine environment. Microb Ecol 49:163–175. doi:10.1007/s00248-003-1057-0. 15688258

[51] Labrenz M, Tindall BJ, Lawson PA, Collins MD, Schumann P, Hirsch P 2000 Staleya guttiformis gen. nov., sp. nov. and Sulfitobacter brevis sp. nov., alpha-3-Proteobacteria from hypersaline, heliothermal and meromictic Antarctic Ekho Lake. Int J Syst Evol Microbiol 50:303–313. doi:10.1099/00207713-50-1-303. 10826817

[52] Moran MA, González JM, Kiene RP 2003 Linking a bacterial taxon to sulfur cycling in the sea: studies of the marine Roseobacter group. Geomicrobiology J 20:375–388. doi:10.1080/01490450303901.

[53] Zachariah S, Kumari P, Das SK 2017 Sulfitobacter pontiacus subsp. fungiae subsp. nov., isolated from coral Fungia seychellensis from Andaman Sea, and description of Sulfitobacter pontiacus subsp. pontiacus subsp. nov. Curr Microbiol 74:404–412. doi:10.1007/s00284-017-1200-7. 28184991

[54] Pukall R, Buntefuss D, Fruhling A, Rohde M, Kroppenstedt RM, Burghardt J, Lebaron P, Bernard L, Stackebrandt E 1999 Sulfitobacter mediterraneus sp. nov., a new sulfite-oxidizing member of the α-Proteobacteria. Int J Syst Bacteriol 49:513–519. doi:10.1099/00207713-49-2-513. 10319472

[55] Buchan A, González JM, Moran MA 2005 Overview of the marine Roseobacter lineage. Appl Environ Microbiol 71:5665–5677. doi:10.1128/AEM.71.10.5665-5677.2005. 16204474 PMC1265941

[56] Zeng Y, Dong P, Qiao Z, Zheng T 2016 Diversity of the aerobic anoxygenic phototrophy gene pufM in Arctic and Antarctic coastal seawaters. Acta Oceanol Sin 35:68–77. doi:10.1007/s13131-016-0877-y.

[57] Kwak M-J, Lee J-S, Lee KC, Kim KK, Eom MK, Kim BK, Kim JF 2014 Sulfitobacter geojensis sp. nov., Sulfitobacter noctilucae sp. nov., and Sulfitobacter noctilucicola sp. nov., isolated from coastal seawater. Int J Syst Evol Microbiol 64:3760–3767. doi:10.1099/ijs.0.065961-0. 25122614

[58] Shiba T 1991 Roseobacter litoralis gen. nov., sp. nov., and Roseobacter denitrificans sp. nov., aerobic pink-pigmented bacteria which contain bacteriochlorophyll a. Syst Appl Microbiol 14:140–145. doi:10.1016/S0723-2020(11)80292-4.

[59] Kalhoefer D, Thole S, Voget S, Lehmann R, Liesegang H, Wollher A, Daniel R, Simon M, Brinkhoff T 2011 Comparative genome analysis and genome-guided physiological analysis of Roseobacter litoralis. BMC Genomics 12:324. doi:10.1186/1471-2164-12-324. 21693016 PMC3141670

[60] Zhong Z-P, Liu Y, Wang F, Zhou Y-G, Liu H-C, Liu Z-P 2015 Lacimonas salitolerans gen. nov., sp. nov., isolated from surface water of a saline lake. Int J Syst Evol Microbiol 65:4550–4556. doi:10.1099/ijsem.0.000611. 26373783

[61] Hameed A, Shahina M, Lin S-Y, Lai W-A, Hsu Y-H, Liu Y-C, Huang Y-M, Young C-C 2013 Shimia biformata sp. nov., isolated from surface seawater, and emended description of the genus Shimia Choi and Cho 2006. Int J Syst Evol Microbiol 63:4533–4539. doi:10.1099/ijs.0.053553-0. 23907225

[62] Hyun D-W, Kim M-S, Shin N-R, Kim JY, Kim PS, Whon TW, Yun J-H, Bae J-W 2013 Shimia haliotis sp. nov., a bacterium isolated from the gut of an abalone, Haliotis discus hannai. Int J Syst Evol Microbiol 63:4248–4253. doi:10.1099/ijs.0.053140-0. 23811138

[63] Chen M-H, Sheu S-Y, Chen CA, Wang J-T, Chen W-M 2011 Shimia isoporae sp. nov., isolated from the reef-building coral Isopora palifera. Int J Syst Evol Microbiol 61:823–827. doi:10.1099/ijs.0.022848-0. 20453104

[64] Choi DH, Cho BC 2006 Shimia marina gen. nov., sp. nov., a novel bacterium of the Roseobacter clade isolated from biofilm in a coastal fish farm. Int J Syst Evol Microbiol 56:1869–1873. doi:10.1099/ijs.0.64235-0. 16902023

[65] Nogi Y, Mori K, Uchida H, Hatada Y 2015 Shimia sagamensis sp. nov., a marine bacterium isolated from cold-seep sediment. Int J Syst Evol Microbiol 65:2786–2790. doi:10.1099/ijs.0.000333. 25977284

[66] Pujalte MJ, Macián MC, Arahal DR, Ludwig W, Schleifer KH, Garay E 2005 Nereida ignava gen. nov., sp. nov., a novel aerobic marine α-proteobacterium that is closely related to uncultured Prionitis (alga) gall symbionts. Int J Syst Evol Microbiol 55:631–636. doi:10.1099/ijs.0.63442-0. 15774635

[67] Arahal DR, Pujalte MJ, Rodrigo-Torres L 2016 Draft genomic sequence of Nereida ignava CECT 5292 T, a marine bacterium of the family Rhodobacteraceae. Stand Genomic Sci 11:1–21. doi:10.1186/s40793-016-0141-2. 26929790 PMC4770636

[68] Harrison PW, Lower RPJ, Kim NKD, Young J 2010 Introducing the bacterial “chromid”: not a chromosome, not a plasmid. Trends Microbiol 18:141–148. doi:10.1016/j.tim.2009.12.010. 20080407

[69] Petersen J, Wagner-Döbler I 2017 Plasmid transfer in the ocean—a case study from the Roseobacter group. Front Microbiol 8:1350. doi:10.3389/fmicb.2017.01350. 28769910 PMC5513947

[70] Bonnett TC, Cobine P, Sockett RE, McEwan AG 1995 Phenotypic characterisation and genetic complementation of dimethylsulfoxide respiratory mutants of Rhodobacter sphaeroides and Rhodobacter capsulatus. FEMS Microbiol Lett 133:163–168. doi:10.1111/j.1574-6968.1995.tb07878.x. 8566702

[71] Addlesee HA, Fiedor L, Hunter CN 2000 Physical mapping of bchG, orf427, and orf177 in the photosynthesis gene cluster of Rhodobacter sphaeroides: functional assignment of the Bacteriochlorophyll synthetase gene. J Bacteriol 182:3175–3182. doi:10.1128/jb.182.11.3175-3182.2000. 10809697 PMC94504

[72] Jones MR, McEwan AG, Jackson JB 1990 The role of c-type cytochromes in the photosynthetic electron transport pathway of Rhodobacter capsulatus. Biochim Biophys Acta 1019:59–66. doi:10.1016/0005-2728(90)90124-m. 2168749

[73] Hahn FM, Baker JA, Poulter CD 1996 Open reading frame 176 in the photosynthesis gene cluster of Rhodobacter capsulatus encodes idi, a gene for isopentenyl diphosphate isomerase. J Bacteriol 178:619–624. doi:10.1128/jb.178.3.619-624.1996. 8550491 PMC177703

[74] Yutin N, Béjà O 2005 Putative novel photosynthetic reaction centre organizations in marine aerobic anoxygenic photosynthetic bacteria: insights from metagenomics and environmental genomics. Environ Microbiol 7:2027–2033. doi:10.1111/j.1462-2920.2005.00843.x. 16309398

[75] Kreiss P, Mailhe P, Scherman D, Pitard B, Cameron B, Rangara R, Aguerre-Charriol O, Airiau M, Crouzet J 1999 Plasmid DNA size does not affect the physicochemical properties of lipoplexes but modulates gene transfer efficiency. Nucleic Acids Res 27:3792–3798. doi:10.1093/nar/27.19.3792. 10481017 PMC148641

[76] Chan V, Dreolini LF, Flintoff KA, Lloyd SJ, Mattenley AA 2002 The effect of increasing plasmid size on transformation efficiency in Escherichia coli. J Exp Microbiol Immunol 2:207–223.

[77] Lassalle F, Périan S, Bataillon T, Nesme X, Duret L, Daubin V 2015 GC-content evolution in bacterial genomes: the biased gene conversion hypothesis expands. PLoS Genet 11:e1004941. doi:10.1371/journal.pgen.1004941. 25659072 PMC4450053

[78] Guillard RRL, Ryther JH 1962 Studies of marine planktonic diatoms: I. Cyclotella nana Hustedt, and Detonula confervacea (Cleve) Gran. Can J Microbiol 8:229–239. doi:10.1139/m62-029. 13902807

[79] Zerbino D, Birney E 2008 Velvet: algorithms for de novo short read assembly using De Bruijn graphs. Genome Res 18:107–829. 10.1101/gr.074492.107 PMC2336801 18349386

[80] Hall TA 1999 BioEdit: a user-friendly biological sequence alignment editor and analysis program for Windows 95/98/NT. Nucleic Acids Symp Ser (41):95–98.

[81] Hall T 2011 BioEdit: an important software for molecular biology. GERF Bull Biosci 2:60–61.

[82] Stamatakis A 2014 RAxML version 8: a tool for phylogenetic analysis and post-analysis of large phylogenies. Bioinformatics 30:1312–1313. doi:10.1093/bioinformatics/btu033. 24451623 PMC3998144

[83] Kumar S, Stecher G, Tamura K 2016 MEGA7: Molecular Evolutionary Genetics Analysis version 7.0 for bigger datasets. Mol Biol Evol 33:1870–1874. doi:10.1093/molbev/msw054. 27004904 PMC8210823

[84] Letunic I, Bork P 2016 Interactive tree of life (iTOL) v3: an online tool for the display and annotation of phylogenetic and other trees. Nucleic Acids Res 44:W242–W245. doi:10.1093/nar/gkw290. 27095192 PMC4987883

