# Supplementary figures and images for: Characteristics and Evolutionary Analysis of Photosynthetic Gene Clusters on Extrachromosomal Replicons: from Streamlined Plasmids to Chromids
Source: mSystems. 2019 Sep 10;4(5):e00358-19. doi: 10.1128/mSystems.00358-19 (PMC6739100; doi:10.1128/mSystems.00358-19)

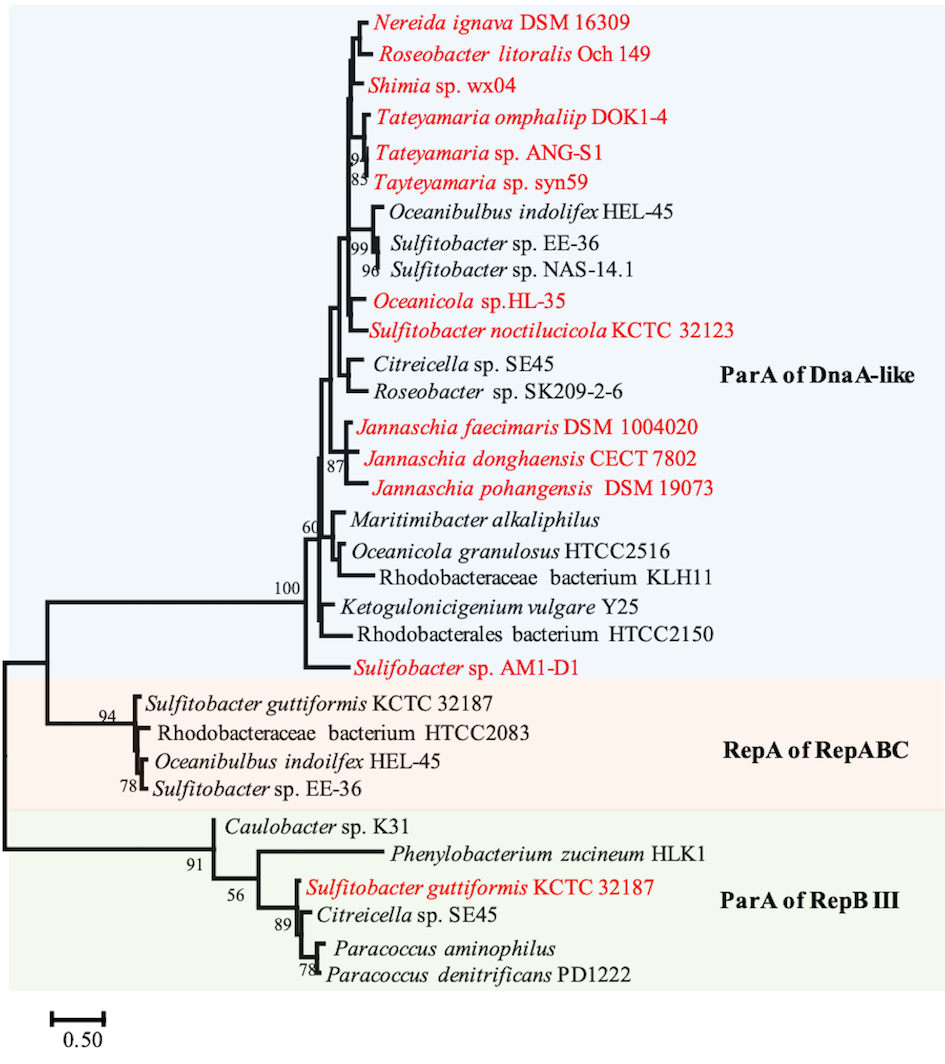

Supplement: FIG S1 [file mSystems.00358-19-sf001.tif]

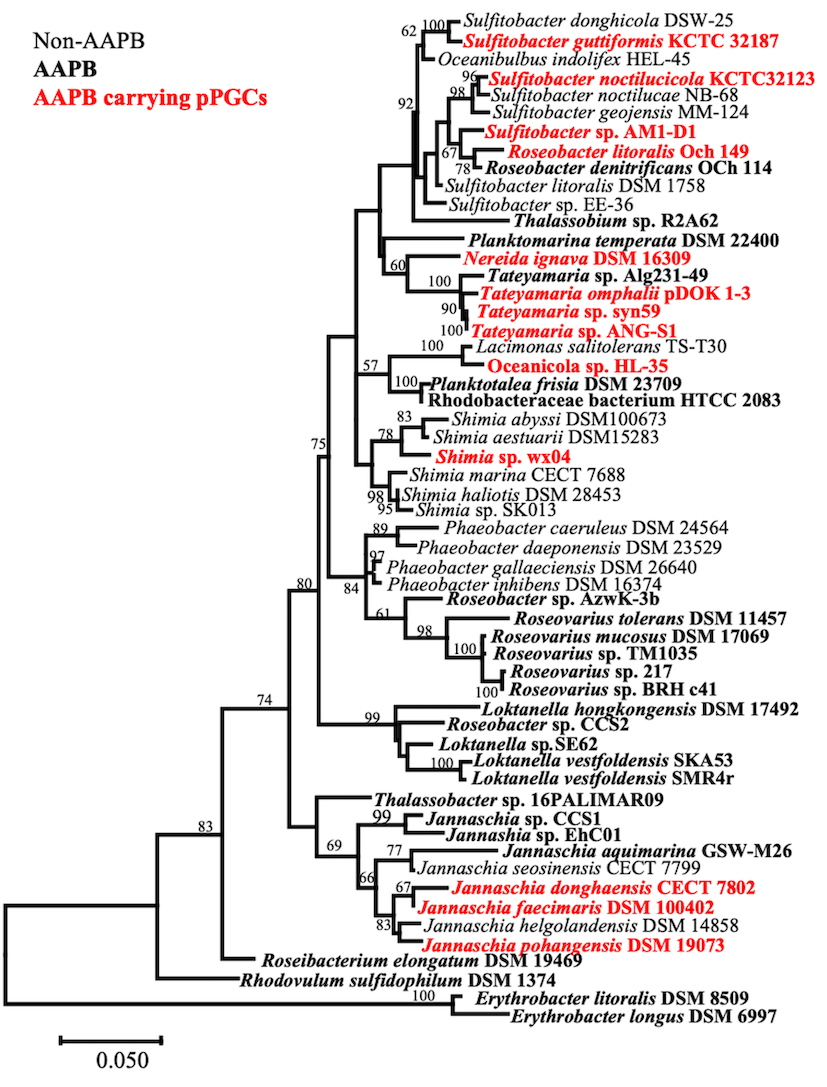

Supplement: FIG S2 [file mSystems.00358-19-sf002.tif]

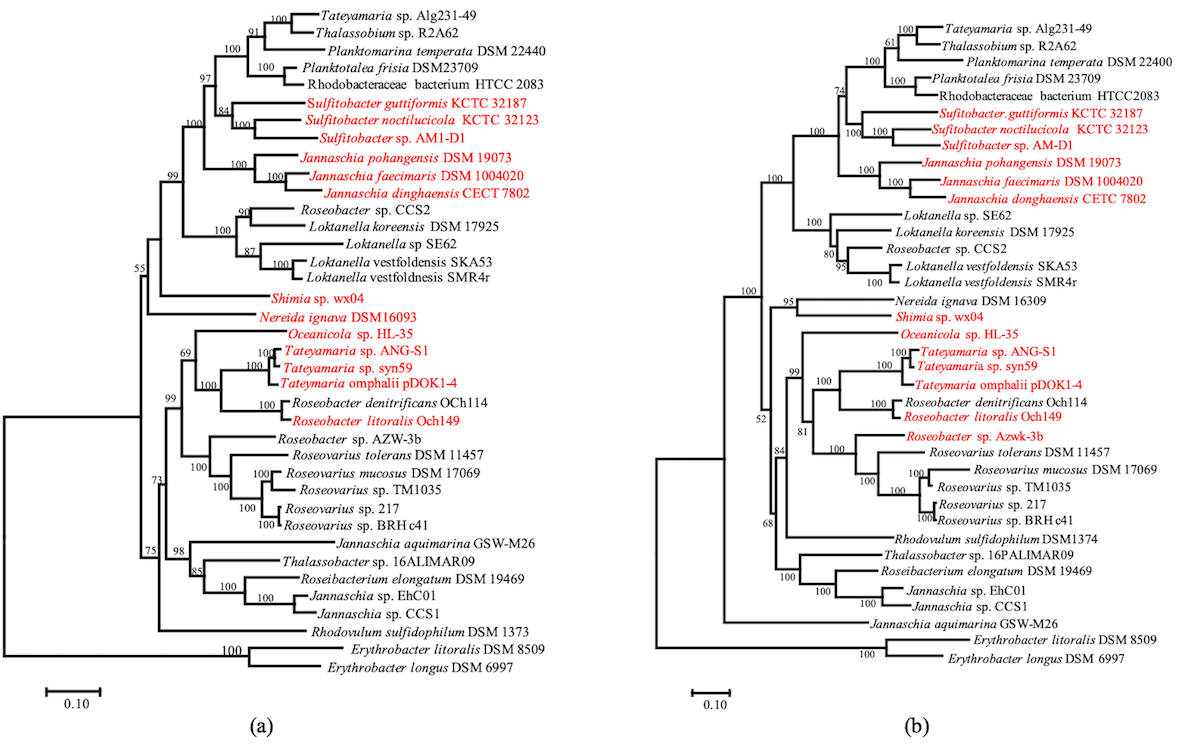

Supplement: FIG S3 [file mSystems.00358-19-sf003.tif]

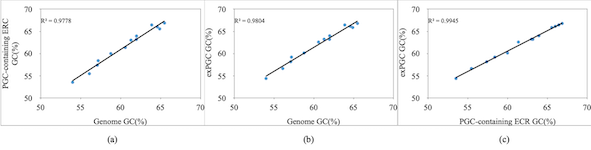

Supplement: FIG S4 [file mSystems.00358-19-sf004.tif]
