# Supplementary material for: Characteristics and Evolutionary Analysis of Photosynthetic Gene Clusters on Extrachromosomal Replicons: from Streamlined Plasmids to Chromids
Source: mSystems. 2019 Sep 10;4(5):e00358-19. doi: 10.1128/mSystems.00358-19 (PMC6739100; doi:10.1128/mSystems.00358-19)
Supplement: TABLE S1 [file mSystems.00358-19-st001.docx]

Table S1

| **ParA gene (Fig. S1):** *Nereida ignava* DSM 16309 (FORZ0100001.1); *Roseobacter litoralis* Och149 (EDQ15589); *Shimia* sp. wx04 (VCDK00000000.1); *Tateyamaria omphaliip* DOK1-4 (CP019315.1); *Tateyamaria* sp. ANG-S1(JWLL01000010.1); *Tateyamaria* sp. syn59 (VCBA00000000.1); *Oceanibulbus indolifex* HEL-45 (EDQ30110); *Sulfitobacter* sp. EE-36 (EAP82888); *Sulfitobacter* sp. NAS-14.1 (EAP79663); *Oceanicola* sp. HL-35 (JAFT01000001.1); *Sulfitobacter noctilucicola* KCTC 32123 (JASD01000002.1); *Citreicella* sp. SE45 (EEX12243); *Jannaschia faecimaris* DSM 1004020 (FNPX01000016.1); *Jannaschia donghaensis* CECT 7802 (CXSU01000001.1); *Jannaschia pohangensis* DSM 19073 (FORA01000009.1); *Maritimibacter alkaliphilus* (EAQ10891); *Oceanicola granulosus* HTCC2516 (EAR51885); Rhodobacteraceae bacterium KLH11(EEE35054); *Ketogulonicigenium vulgare* Y25 (ADO44244); Rhodobacterales bacterium HTCC2150 (EBA01964); *Sulifitobacter* sp. AM1-D1(CP018077); *Sulfitobacter guttiformis* KCTC 32187 (JN172926); Rhodobacteraceae bacterium HTCC2083 (EDZ40476); *Oceanibulbus indoilfex* HEL-45 (ZP_02155162); *Sulfitobacter* sp. EE-36 (EAP82484); *Caulobacter* sp. K31 (ABZ74456); *Phenylobacterium zucineum* HLK1 (ACG79945); *Sulfitobacter guttiformis* KCTC 32187 (JN172927); *Citreicella* sp. SE45 (EEX11939); *Paracoccus aminophilus* (ACY78312); *Paracoccus denitrificans* PD1222 (ABL73095) |
| --- |
| **16S rRNA (Fig. S2)*:*** *Sulfitobacter donghicola* DSW-25(NR_044164.1)*; Sulfitobacter guttiformis* KCTC32187 (JASG01000004.1)*; Oceanibulbus indolifex* HEL*-*45(AJ550939.1)*; Sulfitobacter noctilucicola* KCTC32123 (KC428717.1); *Sulfitobacter geojensis* MM-124 (KC428714.1); *Sulfitobacter* sp. AM1-D1 (KY264919.1); *Roseobacter litoralis* Och149 (CP002623.1); *Roseobacter denitrificans* Och114 (NC_008209.1); *Sulfitobacter litoralis* DSM17584 (NR_043547); *Sulfitobacter* sp. EE-36 (AF007254.2); *Thalassobium* sp. R2A62 (GG697169.2); *Planktomarina temperata* DSM22400 (GQ369962.1); *Nereida ignava* DSM16309 (FORZ01000025.1); *Tateyamaria* sp. Alg231-49 (FRFA01000015.1); *Tateyamaria omphalii* pDOK1-4 (CP019312.1); *Tateyamaria* sp. ANG-S (JWLL01000005.1); *Lacimonas salitolerans* TS-T30 (KC762318.2); *Oceanicola* sp. HL-35 (JAFT01000000.1); *Planktotalea frisia* DSM 23709 (NR_108442.1); Rhodobacteraceae bacterium HTCC2083 (ABXE00000000.1); *Shimia abyssi* DSM100673 (NR_148628); *Shimia aestuarii* DSM15283 (NR_042903.1); *Shimia marina* CECT 7688 (NR_043300.1); *Shimia haliotis* DSM 28453 (NR_109739.1); *Shimia* sp. SK013 (HG423261.2); *Phaeobacter caeruleus* DSM 24564 (NR_118542.1); *Phaeobacter daeponensis* DSM 23529 (NR_044026.1); *Phaeobacter gallaeciensis* DSM26640 (NR_118540.1); *Phaeobacter inhibens* DSM16374 (KC176241.1); *Roseobacter* sp. AzwK-3b (DQ223017.1); *Roseovarius tolerans* DSM11457 (NR_026405.1); *Roseovarius mucosus* DSM 17069 (NR_042159.1); *Roseovarius* sp. TM1035 (ABCL01000012.1); *Roseovarius* sp. 217 (AAMV01000013.1); *Roseovarius* sp. BRH c41 (LADY01000010.1); *Loktanella hongkongensis* DSM17492 (APGJ01000007.1); *Roseobacter* sp. CCS2 (AAYB00000000.1); *Loktanella vestfoldensis* SKA53 (CH672414.1); *Loktanella vestfoldensis* SMR4r (CP021431.1); *Thalassobacter* sp. 16PALIMAR09 (HE981201.1); *Jannaschia* sp. CCS1 (NC_007802.1); *Jannashia* sp. EhC01 (LXYJ01000030.1); *Jannaschia aquimarina* GSW-M26 (NR_109177.1); *Jannaschia seosinensis* CECT7799 (NR_043271.1); *Jannaschia donghaensis* CECT 7802 (NR_044162.1); *Jannaschia helgolandensis* DSM14858 (NR_028976.1); *Jannaschia pohangensis* DSM19073 (NR_043910.1); *Roseibacterium elongatum* DSM 19469 (CP004372.1); *Rhodovulum sulfidophilum* DSM1374 (D16423.1); *Erythrobacter litoralis* DSM8509 (AF465836.1); *Erythrobacter longus* DSM6997 (AF465835.1) |
| **38 bacterial genomes for 16S rRNA and 29 conserved PGC genes concatenation (Fig. 3, Fig. S3):** *Tateyamaria* sp. Alg231-49 (FRFA00000000.1)*; Thalassobium* sp. R2A62 (ACOA00000000.1); *Planktomarina temperata* DSM22400 (CP003984.1); *Planktotalea frisia* DSM23709 (QKZM00000000.1); *Rhodobacteraceae bacterium* HTCC2083 (ABXE00000000.1); *Sulfitobacter guttiformis* KCTC 32187 (ASG00000000.1); *Sulfitobacter noctilucicola* KCTC32123 (JASD00000000.1); *Sulfitobacter* sp. AM1-D1 (CP018076.1); *Jannaschia donghaensis* CECT7802 (CXSU00000000.1); *Jannaschia pohangensis* DSM19073 (FORA00000000.1); *Jannaschia faecimaris* DSM100420 (FNPX00000000.1); *Roseobacter* sp. CCS2 (AAYB00000000.1); *Loktanella vestfoldensis* SMR4r (CP021431.1); *Loktanella vestfoldensis* SKA53 (AAMS00000000.1); *Loktanella* sp. SE62 (PRJNA46507); *Loktanella koreensis* DSM17925 (FOIZ00000000.1); *Nereida ignava* DSM16309 (FORZ00000000.1); *Oceanicola* sp. HL-35 (JAFT00000000.1); *Tateyamaria* sp. ANG-S1 (JWLL00000000.1); *Tateymaria omphalii* pDOK1-4 (CP019319.1); *Roseobacter denitrificans* Och114 (FOOO00000000.1); *Roseobacter* sp. AzwK-3b (ABCR00000000.1); *Roseovarius tolerans* DSM11457 (FOBO00000000.1); *Roseovarius mucosus* DSM17069 (AONH00000000); *Roseovarius* sp. TM1035 (ABCL00000000.1); *Roseovarius* sp. 217 (AAMV00000000.1); *Roseovarius* sp. BRH c41 (LADY00000000.1); *Rhodovulum sulfidophilum* DSM1374 (CP015418.1); *Thalassobacter* sp. 16PALIMAR09 (JHAK00000000.1); *Roseibacterium elongatum* DSM19469 (CP004372.1); *Jannaschia* sp. EhC01 (LXYJ00000000.1); *Jannaschia* sp. CCS1 (CP007802.1); *Jannaschia aquimarina* GSW-M26 (JYFE00000000.1); *Erythrobacter longus* DSM6997(JMIW00000000.1); *Erythrobacter litoralis* DSM8509 (JMIX00000000.1); *Tateyamaria* sp. syn59 (VCBA00000000.1); *Shimia* sp. wx04 (VCDK00000000.1). |
