# Supplementary material for: Characteristics and Evolutionary Analysis of Photosynthetic Gene Clusters on Extrachromosomal Replicons: from Streamlined Plasmids to Chromids
Source: mSystems. 2019 Sep 10;4(5):e00358-19. doi: 10.1128/mSystems.00358-19 (PMC6739100; doi:10.1128/mSystems.00358-19)
Supplement: TABLE S2 [file mSystems.00358-19-st002.docx]

Table S2

| **Strain** | **Extrachromosomal Replicon** | **Type** |
| --- | --- | --- |
| *Tateyamaria* sp. ANG-S1 | pANG-S1-1*^a^* | chromid-like |
|  | pANG-S1-2 | plasmid-like |
|  | pANG-S1-3 | plasmid-like |
|  | pANG-S1-4 | chromd-like |
|  | pANG-S1-5 | chromid-like |
| *Tateyamaria* sp.syn59 | psyn59_1 | chromid-like |
|  | psyn59_2 *^a^* | chromid-like |
|  | psyn59_3 | chromid-like |
|  | psyn59_4 | chromid-like |
|  | psyn59_5 | plasmid-like |
|  | psyn59_6 | chromd-like |
| *Tateyamaria omphalii* DOK1-4 | pDOK1-4-1 | chromid-like |
|  | pDOK1-4-2 | chromid-like |
|  | pDOK1-4-3 *^a^* | chromid-like |
|  | pDOK1-4-4 | chromid-like |
|  | pDOK1-4-5 | plasmid-like |
|  | pDOK1-4-6 | plasmid-like |
|  | pDOK1-4-7 | plasmid-like |
| *Jannaschia faecimaris* DSM1004020 | pJF-1 *^a^* | plasmid-like |
|  | pJF-2 | plasmid-like |
| *Jannaschia pohangensis* DSM19073 | pJP-1 *^a^* | plasmid-like |
|  | pJP-2 | plasmid-like |
| *Jannaschia donghaensis* CECT7802 | pJD-1 *^a^* | plasmid-like |
|  | pJD-1 | plasmid-like |
| *Sulfitobacter* sp. AM1-D1 | pAM1-D1-1 *^a^* | chromid-like |
|  | pAM1-D1-2 | plasmid-like |
|  | pAM1-D1-3 | plasmid-like |
|  | pAM1-D1-4 | chromid-like |
|  | pAM1-D1-5 | plasmid-like |
| *Sulfitobacter noctilucicola* KCTC32123 | pSN-1 | chromid-like |
|  | pSN-2 *^a^* | chromid-like |
|  | pSN-3 | plasmid-like |
|  | pSN-4 | chromid-like |
|  | pSN-5 | plasmid-like |
| *Sulfitobacter guttiformis* KTCT32187 | pSG53 | plasmid-like |
|  | pSG4 | plasmid-like |
|  | pSD118 *^a^* | chromid-like |
| *Roseobacter litoralis* Och149 | pRLO149_63 | plasmid-like |
|  | pRLO149_83 | chromid-like |
|  | pRLO149_94 *^a^* | chromid-like |
| *Oceanicola* sp. HL-35 | pHL-35-1 *^a^* | plasmid-like |
|  | pHL-35-2 | plasmid-like |
| *Shimia* sp. wx04 | pwx04-1-1 | plasmid-like |
|  | pwx04-1-2 *^a^* | plasmid-like |
|  | pwx04-1-3 | plasmid-like |
|  | pwx04-1-4 | chromid-like |
| *Nereida ignava* DSM16309 | pNI *^a^* | chromid-like |

*a* The PGC-containing extrachromosomal replicons are indicated in red
