# Supplementary material for: Characteristics and Evolutionary Analysis of Photosynthetic Gene Clusters on Extrachromosomal Replicons: from Streamlined Plasmids to Chromids
Source: mSystems. 2019 Sep 10;4(5):e00358-19. doi: 10.1128/mSystems.00358-19 (PMC6739100; doi:10.1128/mSystems.00358-19)
Supplement: TABLE S3 [file mSystems.00358-19-st003.docx]

Table S3

| **Accession of *Roseobacter* clade AAPB** *Roseovarius indicus* B108 (LAXI00000000.1); *Roseovarius indicus* EhC03 (LXYQ00000000.1); *Roseovarius mucosus* DSM17069 (AONH00000000.1); *Roseovarius gaetbuli* CECT8370 (FWFJ00000000.1); *Roseovarius* sp. TM1035 (ABCL00000000.1); *Roseovarius tolerans* DSM11457 (FOBO00000000.1); *Roseovarius* sp. AK1035 (CP030099.1); *Roseovarius* sp. 217 (AAMV00000000.1); *Roseovarius* sp. BRH_c41 (LADY00000000.1); *Roseovarius* sp. TE539 (QNGB00000000.1); *Roseovarius azorensis* DSM100674 (FOAG00000000.1)*; Roseovarius confluentis* SAG6 (PPFE00000000.1); *Roseovarius nanhaiticus* DSM29590 (FTNV00000000.1); *Roseovarius nanhaiticus* CGMCC1.10961 (FOAC00000000); *Roseovarius atlanticus*R12B (LAXJ00000000.1); *Loktanella* sp. 5RATIMAR09 (LJAL00000000.1); *Loktanella* sp. DSM 29012 (FOEJ00000000.1); *Loktanella fryxellensis* DSM16213 (FOCI00000000.1); *Loktanella* sp. 3ANDIMAR09(LJAK00000000.1); *Loktanella* sp. PT4BL (QJJL00000000.1); *Marivita hallyeonensis* DSM29431 (FQXC00000000.1); *Loktanella koreensis* DSM17925 (FOIZ00000000.1); *Marivita geojedonensis* DSM29432 (PVTN00000000.1) ; *Marivita cryptomonadis* CL-SK44 (JFKD00000000.1); *Loktanella vestfoldensis* SMR4r (CP021431.1); *Loktanella vestfoldensis* SKA53 (AAMS00000000.1); *Loktanella sp.* SE62 (PRJNA46507); *Tateyamaria* sp. ANG-S1 (JWLL00000000.1); *Tateyamaria omphalii*DOK1-4 (CP019319.1); *Tateyamaria* sp. Alg231-49 (FRFA00000000.1); *Tateyamaria* sp. syn59 (VCBA00000000.1); *Sulfitobacter guttiformis* KCTC 32187 (JASG00000000.1); *Sulfitobacter noctilucicola* KCTC 32123 (JASD00000000.1); *Sulfitobacter* sp. AM1-D1 (CP018076.1); *Jannaschia donghaensis* CECT7802 (CXSU00000000.1); *Jannaschia aquimarina* GSW-M26 (JYFE00000000.1); *Jannaschia* sp. EhC01 (LXYJ00000000.1); *Jannaschia pohangensis* DSM19073 (FORA00000000.1); *Jannaschia faecimaris* DSM 100420 (FNPX00000000.1); *Mameliella alba* JL351 (NIWA00000000.1)*; Jannaschia* sp. CCS1 (NC_007802.1); *Salipiger mucosus* DSM16094 (APVH00000000.1); *Thalassobium* sp. R2A62 (ACOA00000000.1); *Ruegeria profundi*ZGT108 (LQBP00000000.1); *Roseivivax roseus* DSM23042 (FOGU00000000.1); *Roseivivax marinus* JCM19386 (AQQW00000000.1); *Roseivivax halotolerans* JCM10271 (FOXV00000000.1); *Roseivivax lentus* DSM29430 (FOXV00000000.1); *Roseivivax isoporae* LMG 25204 (JAME00000000.1); *Roseivivax atlanticus* 22II (AQQW00000000.1); *Planktomarina temperata* DSM22400 (CP003984); *Litoreibacter ponti* DSM100977 (QBKS00000000.1); *Shimia* sp.wx04 (VCDK00000000.1); *Oceanicola* sp. HL-35 (JAFT00000000.1); *Thalassobacter stenotrophicus* CECT5294 (CYRX00000000.1); *Thalassobacter* sp. 16PALIMAR09 (JHAK00000000.1); *Planktotalea frisia* DSM23709 (QKZM00000000.1); [*Nereida ignava* DSM 16309](https://www.ncbi.nlm.nih.gov/Taxonomy/Browser/wwwtax.cgi?id=282199) (FORZ00000000.1); *Phaeobacte*r sp. 22II1-1F12B (AQQP00000000.1); *Pelagicola* sp. LXJ1103 (QFAR00000000.1); *Roseobacter litoralis* Och 149 (NC_008209.1); *Roseobacter denitrificans* OCh114 (NC_008209.1); *Roseobacter* sp. AzwK-3b (ABCR00000000.1); *Roseobacter* sp. CCS2 (AAYB00000000.1); *Pontivivens insulae* CECT8812 (OMKW00000000.1); *Pontivivens insulae* DSM103361 (QRDO00000000.1); *Roseisalinus antarcticus*CECT7023 (FWFZ00000000.1); *Ponticoccus* sp. LZ-14 (RBVZ00000000.1); *Thalassococcus* sp. WRAS1 (QPMK00000000.1) |
| --- |
