# Supplementary material for: Characteristics and Evolutionary Analysis of Photosynthetic Gene Clusters on Extrachromosomal Replicons: from Streamlined Plasmids to Chromids
Source: mSystems. 2019 Sep 10;4(5):e00358-19. doi: 10.1128/mSystems.00358-19 (PMC6739100; doi:10.1128/mSystems.00358-19)
Supplement: TABLE S4 [file mSystems.00358-19-st004.docx]

| **Gene** | **Functions role** | **Biological process** |
| --- | --- | --- |
| *bchI* | Mg-chelatase subunit I | Bacteriochlorophyll biosynthesis |
| *bchD* | Mg-chelatase subunit D | Bacteriochlorophyll biosynthesis |
| *bchO* | Putative hydrolase | Bacteriochlorophyll biosynthesis |
| *tspO* | Tryptophan-rich sensory protein | Ttrapyrrole metabolism |
| *crtC* | Hydroxyneurosporene dehydrogenase | Carotenoid biosynthesis |
| *crtD* | Methoxyneurosporene dehydrogenase | Carotenoid biosynthesis |
| *crtF* | Hydroxynuerosporene-O-methyltransferase | Carotenoid biosynthesis |
| *bchC* | 3-Hydroxyethyl bacterochlorophyl ester oxidative | Light-independent bacteriochlorophyll biosynthesis |
| *bchX* | Bacteriochlorophyllide reductase, subunit X | Bacteriochlorophyll biosynthesis |
| *bchY* | Bacteriochlorophyllide reductase, subunit Y | Light-independent bacteriochlorophyll biosynthesis |
| *bchZ* | Bacteriochlorophyllide reductase, subunit Z | bacteriochlorophyll biosynthesis |
| *pufL* | Photosynthetic reaction center L subunit | Photosynthetic electron transport in photosystem II |
| *pufM* | Photosynthetic reaction center M subunit | Photosynthetic electron transport in photosystem II |
| *bchP* | Geranylgeranyl hydrogenase | Light-independent bacteriochlorophyll biosynthesis |
| *puCC* | Putative LH assembly protein | LH assembly |
| *bchG* | Bacteriochlorophyll synthase | Light-independent bacteriochlorophyll biosynthesis |
| *ppSR* | Transcriptional regulator PpsR | Transcriptional regulator PpsR |
| *bchF* | 2-vinyl bacteriochlorophyllide hydratase | Light-independent bacteriochlorophyll biosynthesis |
| *bchN* | Protochlorophyllide reductase, subunit N | Light-independent bacteriochlorophyll biosynthesis |
| *bchB* | Protochlorophyllide reductase, subunit B | Light-independent bacteriochlorophyll biosynthesis |
| *bchH* | Mg-chelatase subunit H | Light-independent bacteriochlorophyll biosynthesis |
| *bchL* | Protochlorophyllide reductase (DPOR), subunit L | Light-independent bacteriochlorophyll biosynthesis |
| *bchM* | Mg-protoporphyrin IX SAM O-methyltransferase | Light-independent bacteriochlorophyll biosynthesis |
| *IhaA* | Possible assembly factor of LH1 | ­LH assembly |
| *puhA* | Photosynthetic reaction center H subunit | LH assembly |
| *puhB* | Possible assembly factor of RC | LH assembly |
| *puhC* | Possible reorganization factor of RC/LH1 | LH assembly |
| *ascF* | Mg protoporphyrin IX monomethyl ester oxidative cyclase (aerobic) | Bacteriochlorophyll biosynthesis |
| *puhE* | Possible assembly factor of RC/LH1 | LH assembly |

Table S4
